# Single versus tandem autologous stem cell transplantation in newly diagnosed multiple myeloma

**DOI:** 10.1038/s41409-024-02490-1

**Published:** 2024-12-05

**Authors:** Nora Grieb, Alexander Oeser, Maximilian Ferle, Franziska Hanke, Sarah Flossdorf, Sandra Sauer, Hartmut Goldschmidt, Carsten Müller-Tidow, Hans-Jürgen Salwender, Roland Fenk, Monika Engelhardt, Robert Zeiser, Vladan Vucinic, Georg-Nikolaus Franke, Igor Wolfgang Blau, Daniel Teschner, Hermann Einsele, Christoph Kimmich, Miriam Kull, Britta Besemer, Nico Gagelmann, Nicolaus Kröger, Thomas Neumuth, Uwe Platzbecker, Maximilian Merz, on behalf of the German Registry for Hematopoietic Stem Cell Transplantation and Cell Therapy (DRST), Franziska Hanke, Sarah Flossdorf, Sandra Sauer, Hans-Jürgen Salwender, Roland Fenk, Robert Zeiser, Igor Wolfgang Blau, Daniel Teschner, Christoph Kimmich, Miriam Kull, Britta Besemer, Nicolaus Kröger, Uwe Platzbecker

**Affiliations:** 1 Innovation Center Computer Assisted Surgery (ICCAS), University of Leipzig, Leipzig, Germany; 2 Department of Hematology, Hemostaseology, Cellular Therapy and Infectiology, University Hospital Leipzig, Leipzig, Germany; 3 Center for Scalable Data Analytics and Artificial Intelligence (ScaDS.AI) Dresden/Leipzig, University of Leipzig, Leipzig, Germany; 4 German Registry for Hematopoietic Stem Cell Transplantation and Cell Therapy (DRST), Ulm, Germany; 5 Institute for Medical Informatics, Biometry and Epidemiology, University Hospital, University Duisburg-Essen, Duisburg, Germany; 6 Department of Internal Medicine V, University Hospital Heidelberg, Heidelberg, Germany; 7 GMMG Study Group at Department of Internal Medicine V, University Hospital Heidelberg, Heidelberg, Germany; 8 Asklepios Tumor Center Hamburg, AK Altona and AK St Georg, Hamburg, Germany; 9 Department of Hematology, Oncology and Clinical Immunology, Heinrich-Heine University, Düsseldorf, Germany; 10 Department of Hematology, Oncology and Stem Cell Transplantation, University of Freiburg, Freiburg, Germany; 11 Department for Haematology, Oncology and Tumorimmunology, Medical Clinic, Charité University Medicine Berlin, Berlin, Germany; 12 Department of Internal Medicine II, University Hospital Würzburg, Würzburg, Germany; 13 Department of Oncology and Hematology, University Hospital Oldenburg, Oldenburg, Germany; 14 Internal Medicine III, University Hospital Ulm, Ulm, Germany; 15 Department of Hematology, Oncology, Immunology, and Rheumatology, University Hospital Tübingen, Tübingen, Germany; 16 Department for Stem Cell Transplantation, University Cancer Centre Hamburg-Eppendorf, Hamburg, Germany

**Keywords:** Myeloma, Medical research

## Abstract

Identifying patients who may benefit from autologous stem cell transplantation (ASCT) in newly diagnosed multiple myeloma is crucial, especially in the era of effective induction and consolidation strategies. We analyzed data from 12763 patients enrolled in the German Registry for Hematopoietic Stem Cell Transplantation and Cell Therapy (DRST), distinguishing those who underwent single (*n* = 8736) or tandem ASCT (*n* = 4027) from 1998 to 2021. Our findings show that the median age at first ASCT increased over time, while the use of tandem ASCT declined. The shift in treatment practices coincided with higher rates of complete response (CR) post-induction therapy. Significantly improved overall survival and event-free survival over time were observed across all age groups, especially in older patients, but not in patients under 40. Tandem ASCT showed benefits for patients who did not achieve CR after initial ASCT. However, patients with ISS III and renal impairment had poorer outcomes with tandem ASCT. In conclusion, while ASCT remains an important anti-myeloma tool, careful patient selection for tandem ASCT is essential, particularly avoiding its use in patients with ISS III and renal impairment, older age, and those already achieving CR after initial ASCT.

## Introduction

High-dose chemotherapy (HDT) with melphalan followed by autologous stem cell transplantation (ASCT) is still a standard of care for fit patients with newly diagnosed multiple myeloma (NDMM). With every new drug approved for the treatment of NDMM, the role of ASCT is challenged. Nevertheless, even in the era of quadruplet induction therapies with anti-CD38 antibodies in combination with proteasome inhibitors (PI), immunomodulatory drugs (IMiDs) and steroids, ASCT plays an integral role to deepen responses that last for years or even decades [1–5].

Despite its frequent application in NDMM patients, there are several areas of uncertainty connected to ASCT. Identifying patients who benefit the most from this invasive treatment modality remains challenging. The evolution of modern induction therapies led to unprecedented rates of deep, long-lasting remissions that can be achieved even without the application of HDT and ASCT. Therefore, it is important to analyze the impact of ASCT in patients based on their response to induction therapy over time.

Treatment of patients with high-risk disease represents another challenge, even in the era of novel agents [6]. In the past, it has been proposed that the application of tandem ASCT, i.e., a second ASCT within 6 months after the first application improves the outcome of high-risk patients [7]. However, most of the clinical trials that compared single with tandem ASCT were performed before the introduction of modern induction therapies, thus not taking into accont the impact of improved remissions before ASCT [8, 9].

Multiple studies demonstrated that ASCT can be performed safely even beyond the age of 70 years in select patients [10, 11]. However, younger patients with NDMM represent a cohort with special challenges [12]. Since HDT with melphalan increases the lifetime risk for secondary primary malignancies [13], it is important to analyze whether all age groups benfit from ASCT and whether the improved remission rates before ASCT in recent years translated into prolonged survival regardless of age.

To answer the raised question, we analyzed data from the German Registry for Hematopoietic Stem Cell Transplantation and Cell Therapy (DRST), one of the largest registries world-wide to document outcomes of NDMM patients after ASCT.

## Materials and methods

### Data selection

**Fig. 1:**
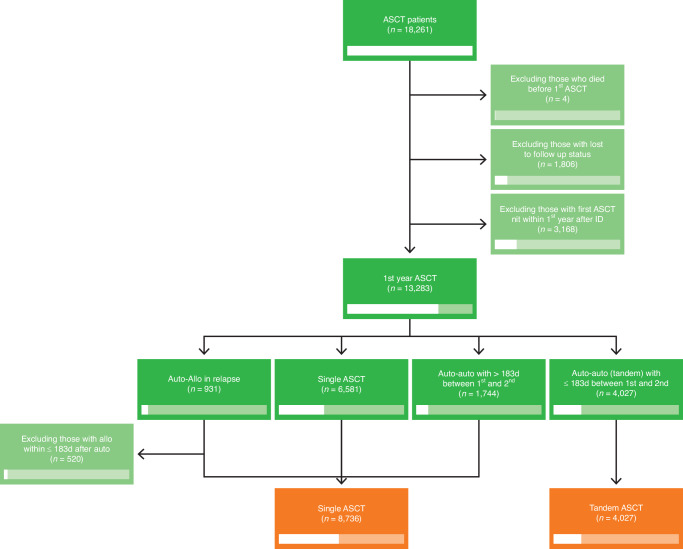
Selection process and classification of cases included in the study.

We included 12763 NDMM patients from 94 centers, who received an ASCT between 1998 and 2021. Data collection and analysis was approved by ethics committees of participating DRST centers and the study was performed according to the declaration of Helsinki. We excluded those who died before the first ASCT, those whose status was reported as lost to follow-up, and those who did not receive the first ASCT within one year after initial diagnosis (ID). We defined tandem transplantation as two ASCTs within 6 months and compared this cohort to patients who did not receive a second transplant and patients who did receive a second transplant (autologous or allogeneic), but not within the 6 months after the first ASCT (Fig. 1). A list of centers and number of patients included is listed in Supplementary Table 1.

Remission before ASCT was measured after induction therapy and specified according to the European Bone Marrow Transplantation (EMBT) and the International Myeloma Working Group (IMWG) [14, 15]. Patients with Salmon-Durie Staging B were classified as having a renal impairment [16].

### Statistical analyses

Overall survival (OS) was defined as the time between first ASCT and death, event free survival (EFS) was defined as the time from first ASCT to either progressive disease (PD), relapse or death. We defined early relapse (ER) as PD or death within 12 months of the first ASCT. Due to major shifts in treatment regimens with the introduction of Bortezomib-based induction therapies in 2008 and Lenalidomide maintenance therapy in 2017 in Germany, we stratified our analyses to 1998–2007, 2008–2016, and 2017–2021. We performed the chi-squared test for categorical and the Kruskall-Wallis test for continuous variables when comparing patient characteristics. Survival probabilities were calculated with the Kaplan-Meier method. Proportional hazards (PH) assumptions were checked based on the scaled Schoenfeld residuals, and if supported, the log-rank test was applied for comparison between subgroups. When comparing transplantation strategies, we accounted for immortal time bias by performing a landmark analysis including only patients with EFS within the first 6 months after initial ASCT. Multivariable Cox PH regression analysis was performed and *p* < 0.05 were considered statistically significant. Statistical analysis was carried out with R version 4.3.2, packages used are listed in Supplementary Table 2.

## Results

### Patient characteristics

We analyzed 12,763 patients who were newly diagnosed between the years of 1998 to 2021 and received an ASCT within the first year after ID. Patients underwent either tandem ASCT (*n* = 4027) or single ASCT, which includes those who underwent only one ASCT (*n* = 6581), as well as those who received a following ASCT or alloSCT after the first 6 months of the first ASCT (*n* = 1744 and *n* = 411, respectively). The median age at first ASCT increased from 59.13 before 2008 to 61.36 years after 2017 (*p* < 0.001). Furthermore, there was a shift in transplantation practices over time, with tandem ASCT becoming less common in recent years (47.4% before 2008 to 25.7% after 2017, *p* < 0.001). All patient characteristics for the respective time periods are summarized in Table 1.

**Table 1:** Patient characteristics.

Feature	Characteristic	1998–2007 (*N* = 3233)	2008–2016 (*N* = 6610)	2017–2021 (*N* = 2920)	*p*-value
Sex (NA = 9)	Female	1323 (40.9%)	2563 (38.8%)	1148 (39.4%)	*p* = 0.08
	Male	1910 (59.1%)	4042 (61.2%)	1768 (60.6%)	
t(4;14) (NA = 0)	yes	9 (0.3%)	269 (4.1%)	342 (11.7%)	*p* < 0.001
	no/not done	3224 (99.7%)	6341 (95.9%)	2578 (88.3%)	
t(14;16) (NA = 0)	yes	0 (0%)	29 (0.4%)	41 (1.4%)	*p* < 0.001
	no/not done	3233 (100%)	6581 (99.6%)	2879 (98.6%)	
del(17p) (NA = 0)	yes	0 (0%)	31 (0.5%)	139 (4.8%)	*p* < 0.001
	no/not done	3233 (100%)	6579 (99.5%)	2781 (95.2%)	
ampl(1q) (NA = 0)	yes	1 (0%)	159 (2.4%)	225 (7.7%)	*p* < 0.001
	no/not done	3232 (100%)	6451 (97.6%)	2695 (92.3%)	
ISS (NA = 8761)	I	18 (41.9%)	683 (39.4%)	743 (33.4%)	*p* < 0.001
	II	14 (32.6%)	531 (30.6%)	811 (36.4%)	
	III	11 (25.6%)	520 (30%)	671 (30.2%)	
Karnofsky (NA = 2997)	100	290 (27.9%)	1535 (25.4%)	780 (29.1%)	*p* < 0.001
	90	487 (46.8%)	3081 (51%)	1225 (45.7%)	
	80	190 (18.3%)	1127 (18.6%)	580 (21.6%)	
	70	68 (6.5%)	234 (3.9%)	69 (2.6%)	
	<=60	5 (0.5%)	66 (1.1%)	29 (1.1%)	
Heavy chain (NA = 4602)	IgA	543 (25.4%)	1036 (24.8%)	479 (25.9%)	*p* = 0.13
	IgG	1560 (73%)	3072 (73.6%)	1339 (72.5%)	
	Others	35 (1.6%)	67 (1.6%)	30 (1.6%)	
Light chain (NA = 875)	Both	3 (0.1%)	20 (0.3%)	2 (0.1%)	*p* < 0.001
	Kappa	1741 (65.6%)	4167 (65.1%)	1843 (65%)	
	Lambda	909 (34.3%)	2212 (34.6%)	991 (34.9%)	
Status after induction therapy (NA = 867)	CR	220 (7.4%)	759 (12.3%)	354 (13%)	*p* < 0.001
	VGPR	20 (0.7%)	1733 (28%)	1105 (40.4%)	
	PR	2073 (69.8%)	3033 (49%)	1047 (38.3%)	
	SD	523 (17.6%)	475 (7.7%)	184 (6.7%)	
	PD	136 (4.6%)	191 (3.1%)	43 (1.6%)	
Status after 1st ASCT (NA = 2085)	CR	518 (20.5%)	1702 (29.7%)	785 (32.3%)	*p* < 0.001
	Never in CR	2006 (79.5%)	4022 (70.3%)	1645 (67.7%)	
Renal Impairment (NA = 0)	yes	541 (16.7%)	1212 (18.3%)	492 (16.8%)	*p* = 0.07
	no	2692 (83.3%)	5398 (81.7%)	2428 (83.2%)	
Regime (NA = 0)	single	1110 (34.3%)	3452 (52.2%)	2019 (69.1%)	*p* < 0.001
	tandem	1533 (47.4%)	1743 (26.4%)	751 (25.7%)	
	double	475 (14.7%)	1158 (17.5%)	111 (3.8%)	
	allo	115 (3.6%)	257 (3.9%)	39 (1.3%)	
Cause of Death (NA = 8981)	No deaths during FU	1092 (33.8%)	4083 (61.8%)	2567 (87.9%)	*p* < 0.001
	HSCT related	182 (5.6%)	211 (3.1%)	25 (0.9%)	
	Relapse/PD	1407 (43.5%)	1604 (24.3%)	206 (7.1%)	
	Secondary malignancy	48 (1.5%)	84 (1.3%)	15 (0.5%)	
	other/unknown	504 (15.6%)	628 (9.6%)	107 (3.7%)	
Cond. Dose (NA = 3694)	100 mg/m^2^	86 (9.3%)	278 (5.1%)	75 (2.8%)	*p* < 0.001
	140 mg/m^2^	114 (12.3%)	1085 (19.8%)	539 (20.3%)	
	200 mg/m^2^	726 (78.4%)	4130 (75.2%)	2036 (76.8%)	
Age at first ASCT (NA = 0)	Median	59.13	60.53	61.36	*p* < 0.001
	IQR	12.02	11.81	10.98	
Days to first ASCT (NA = 0)	Median	197	180	191	*p* < 0.001
	IQR	83	68	70	

### Outcome trends across age groups

**Fig. 2:**
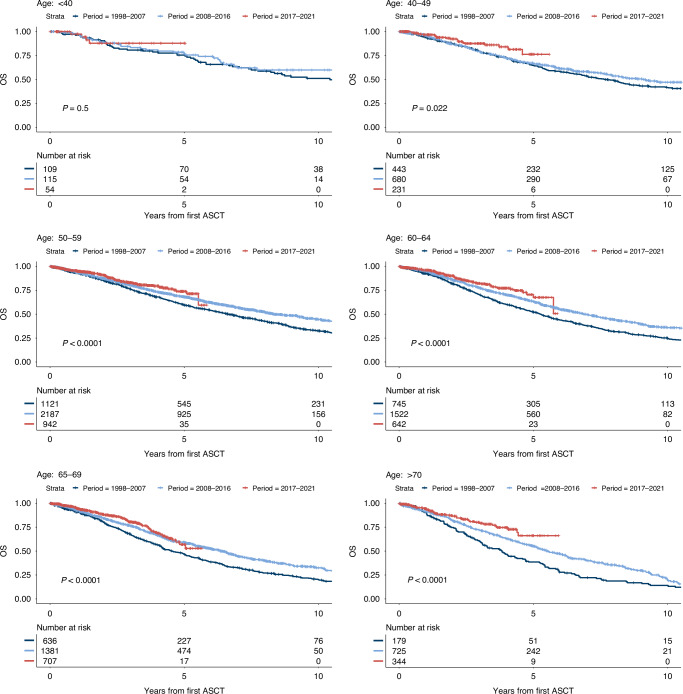
Overall survival from first ASCT by age group and time period.

In a next step, we investigated whether the improved remission before and after ASCT in recent decades translated into prolonged survival, regardless of age. Figure 2 demonstrates significantly improved OS rates across different time periods for all age groups except for very young MM patients under 40. Analysis of two and 5-year OS and EFS rates, as presented in Table 2, further supported these findings. Notably, patients under 40 years of age at ID exhibited stable 2-year survival rates ranging from 90.5% to 87.8%, with substantial improvements in 5-year rates ranging from 75.5% to 88.8%. Similarly, the 2-year EFS rates ranged 67.7% to 74.6% and the 5-year EFS rates from to 36.7% to 42.8%. 2017–2021 median EFS is highest in the 40–49 and <40 age cohort with 4.09 and 4.08 years, respectively and decreases with higher age to 2.78 year in patients >70 years of age. While there was a positive trend in 2-year and 5-year OS and EFS in all age groups, the biggest improvement was seen in patients >70 with a 2-year EFS improving 33.6%, from 35.3% in the 1998–2007 time period to 69.1% in 2017–2021 and the 5-year OS improving 27.6% from 38.6 in 1998–2007 to 66.16% after 2017. This positive survival trend especially in older individuals was accompanied by increased usage of ASCT. While in the time period before 2007 only 5.5% of ASCTs were performed in patients in their seventies, the proportion increased to 11.8% after 2017.

**Table 2:** OS and EFS estimates by age group and time period. If no median is calculated, <50% of patients had an event at time of assessment.

Age	Time Period	Outcome	2-year percentage		5-year percentage		Median (years)
<40	1998–2007	OS	90.51	(85.08–96.29)	75.45	(67.51–84.33)	10.4
		EFS	71.42	(63.27–80.63)	36.40	(28.01–47.3)	3.52
	2008–2016	OS	89	(83.07–95.35)	76.96	(68.67–86.25)	-
		EFS	67.73	(59.09–77.63)	42.80	(33.61–54.51)	3.81
	2017–2021	OS	87.8	(77.17–99.89)	87.8	(77.17–99.89)	-
		EFS	74.64	(61.01–91.32)	39.19	(18.98–80.90)	4.08
40–49	1998–2007	OS	87.26	(84.08–90.56)	64.7	(60.07–69.69)	7.64
		EFS	62.27	(57.69–67.22)	31.34	(26.96–36.43)	2.93
	2008–2016	OS	86.78	(84.09–89.57)	66.89	(62.98–71.04)	9.05
		EFS	57.97	(54.08–62.14)	27.4	(23.81–31.53)	2.52
	2017–2021	OS	92.3	(88.14–96.65)	76.26	(64.58–90.06)	-
		EFS	74.52	(67.65–82.08)	36.7	(23.52–57.3)	4.09
50–59	1998–2007	OS	85.21	(83.07–87.4)	59.4	(56.38–62.57)	6.63
		EFS	58.56	(55.61–61.67)	27.47	(24.77–30.46)	2.5
	2008–2016	OS	87.22	(85.7–88.77)	68.02	(65.78–70.33)	8.35
		EFS	58.84	(55.61–61.67)	25.79	(23.73–28.03)	2.52
	2017–2021	OS	90.73	(88.42–93.11)	73.78	(68.11–79.92)	-
		EFS	73.63	(70.08–77.36)	41.07	(34.94–48.29)	3.87
60–64	1998–2007	OS	81.67	(78.8–84.65)	52.18	(48.4–56.25)	5.25
		EFS	53.29	(49.60–57.24)	21.32	(24.31–29.58)	1.99
	2008–2016	OS	85.58	(83.65–87.56)	63.24	(60.43–66.19)	6.92
		EFS	58.96	(56.28–61.78)	26.82	(19.32–24.63)	2.36
	2017–2021	OS	90.02	(87.1–93.04)	67.49	(58.51–77.85)	-
		EFS	67.98	(63.35–72.94)	36.62	(28.28–47.42)	3.05
65–69	1998–2007	OS	79.99	(76.75–83.36)	46.75	(42.64–51.25)	4.54
		EFS	48.69	(45.70–54.03)	18.16	(15.13–21.8)	1.82
	2008–2016	OS	84.14	(82.04–86.3)	59.16	(56.14–62.34)	6.34
		EFS	57.37	(54.51–60.37)	21.82	(19.32–24.63)	2.24
	2017–2021	OS	88.3	(85.33–91.37)	56.86	(48.01–67.35)	-
		EFS	71.67	(67.4–76.21)	29.31	(20.82–41.27)	2.91
>=70	1998–2007	OS	74.68	(68.17–81.81)	38.57	(31.36–47.45)	3.83
		EFS	35.31	(28.49–43.78)	11.12	(7.03–17.6)	1.33
	2008–2016	OS	81.99	(78.99–85.1)	55.16	(51.07–59.58)	5.53
		EFS	52.91	(49.07–57.06)	19.79	(16.66–23.52)	1.97
	2017–2021	OS	86.52	(82.11–91.17)	66.16	(55.78–78.47)	-
		EFS	69.14	(63.14–75.70)	29.99	(20.96–42.9)	2.78

### Multivariable analysis

Since we saw significant differences in improved survival rates across different age groups in recent years, we fitted multivariable Cox PH models to evaluate the impact of patient and treatment characteristics on OS and EFS across transplantation protocols. In the multivariable analysis presented in Table 3, nonCR status following the initial ASCT was associated with poorer OS and EFS among patients undergoing single ASCT (HR 1.5, 95% CI 1.2–1.86, *p* < 0.001 and HR 1.31, 95% CI 1.14–1.51, *p* < 0.001, respectively). Additionally, failure to achieve CR after induction therapy was linked to shorter EFS in single ASCT patients (HR 1.26, 95% CI 1.04–1.51, *p* = 0.02). No significant associations between remission status post-induction or post-ASCT and either OS or EFS were observed in patients receiving a tandem ASCT. Furthermore, among patients receiving single upfront ASCT, lower Karnofsky performance status was correlated with increased risks of mortality and disease progression. Additionally, the multivariable analyses identified several factors associated with improved outcomes across both single and tandem transplantation approaches. These included younger age at transplantation, absence of t(4;14) translocation, ISS I, IgG as the involved heavy chain, and ID in more recent time periods.

**Table 3:** Multivariable Cox PH analysis on OS and EFS, stratified for transplantation regime.

Factor	Level	Regime	OS	CI	*p*	EFS	CI	*p*
			HR			HR		
Age		single	1.02	1.01–1.03	0.001**	1	1–1.01	0.26
		tandem	1.04	1.02–1.06	<0.001***	1.01	1–1.03	0.03
Body Mass Index		single	0.98	0.98–1.01	0.81	1.01	1–1.02	0.20
		tandem	0.97	0.94–1	0.99	0.99	0.97–1.01	0.47
Sex	female		1			1		
	male	single	1.04	0.89–1.23	0.6	1.09	0.97–1.22	0.15
		tandem	0.98	0.73–1.31	0.87	0.94	0.77–1.15	0.54
t(4;14)	no		1			1		
	yes	single	1.39	0.91–2.12	0.12	1.55	1.16–2.07	0.003**
		tandem	2.05	1.1–3.84	0.02*	1.65	1.09–2.5	0.02*
t(14;16)	no		1			1		
	yes	single	0.68	0.21–2.17	0.51	0.88	0.4–1.96	0.76
		tandem	4.22	0.45–39.52	0.21	2.17	0.61–7.69	0.23
del(17p)	no		1			1		
	yes	single	1.13	0.57–2.23	0.73	1.51	0.94–2.42	0.89
		tandem	0.66	0.25–1.69	0.38	0.48	0.23–0.99	0.05*
ampl(1q)	no		1			1		
	yes	single	0.84	0.54–1.31	0.44	1.02	0.76–1.37	0.9
		tandem	1.31	0.71–2.42	0.38	1.22	0.8–1.86	0.35
ISS	I		1			1		
	II	single	1.52	1.19–1.93	<0.001***	1.31	1.14–1.51	0.004**
		tandem	2	1.27–3.16	0.003**	1.44	1.08–1.91	0.01*
	III	single	1.86	1.45–2.39	<0.001***	1.26	1.04–1.51	<0.001***
		tandem	2.25	1.38–3.66	0.001**	1.87	1.37–2.54	<0.001***
Heavy Chain	IgA		1			1		
	IgG	single	0.73	0.59–0.9	0.003**	0.76	0.66–0.88	<0.001***
		tandem	0.74	0.5–1.08	0.12	0.76	0.58–1	0.05*
Light Chain	Kappa		1			1		
	Lambda	single	1.07	0.91–1.26	0.41	1.07	0.95–1.2	0.27
		tandem	1.25	0.92–1.71	0.16	1.18	0.95–1.47	0.12
Remission after induction therapy	CR		1			1		
	nonCR	single	1.2	0.91–1.57	0.18	1.26	1.04–1.51	0.02*
		tandem	1.45	0.81–2.6	0.22	1.31	0.88–1.95	0.18
Remission after 1st ASCT	CR		1			1		
	nonCR	single	1.5	1.2–1.86	<0.001***	1.31	1.14–1.51	<0.001***
		tandem	1	0.5–2.03	0.99	0.97	0.6–1.55	0.47
Renal Impairment	no		1			1		
	yes	single	0.87	0.7–1.08	0.21	0.94	0.81–1.09	0.4
		tandem	1.36	0.93–2.01	0.12	1.13	0.84–1.52	0.41
Karnofsky		single	0.98	0.97–0.99	<0.001***	0.99	0.99–1	0.008**
		tandem	0.9	0.97–1.01	0.22	1	0.99–1.01	0.81
Conditioning Dose	100		1			1		
	140	single	0.94	0.61–1.45	0.78	0.95	0.7–1.3	0.76
		tandem	1.2	0.64–2.24	0.57	1.26	0.82–1.94	0.29
	200	single	0.92	0.61–1.39	0.68	0.92	0.68–1.24	0.58
		tandem	0.97	0.55–1.72	0.93	0.92	0.62–1.36	0.67
Time Period	1998–2007		1			1		
	2008–2016	single	0.48	0.32–0.72	<0.001***	0.67	0.47–0.95	0.02*
		tandem	0.54	0.34–0.88	0.01*	0.73	0.49–1.08	0.11
	2017–2021	single	0.43	0.28–0.65	<0.001***	0.5	0.35–0.71	<0.001***
		tandem	0.48	0.28–0.83	0.009**	0.48	0.31–0.72	<0.001***

### Benefit of tandem ASCT based on patient and disease characteristics

**Fig. 3:**
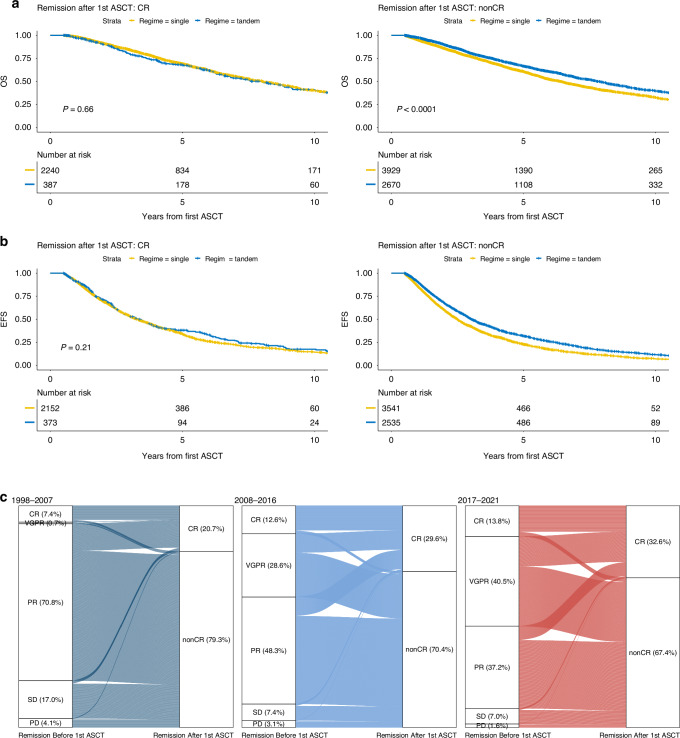
Benefit of tandem transplantation for remission after ASCT based on a 6 month landmark analysis. Comparisons are shown in OS (**a**), in EFS (**b**), and of remission rates pre- and post ASCT across different time periods (**c**).

In recent years, a decline in patients receiving tandem ASCT was observed in Germany, while the rates of deep remissions before ASCT increased significantly. Therefore, we compared OS and EFS between patients who underwent single or tandem ASCT and found no significant differences in patients who achieved CR after the first transplantation compared to others (*p* = 0.66 for OS, *p* = 0.21 for EFS). However, a significant benefit was noted in patients receiving tandem ASCT who did not achieve CR after the initial ASCT (*p* < 0.001 for OS, proportional hazard assumptions not supported for EFS, see Fig. 3a, b). The significant benefit of tandem over single ASCT was also seen in patients who did not achieve CR after induction therapy (Supplementary Fig. 1). Due to data availability, the analysis VGPR or better vs. PR or worse was only feasible in the patients treated after 2006 and in the treatment stage after induction therapy. We found that those patients in VGPR after induction therapy still benefitted from a tandem transplantation in terms of OS and EFS (*p* = 0.049 and *p* < 0.001 respectively). In Fig. 3c, the achieved remission states after induction therapy and the improvement by a first ASCT within the different time periods are shown. The transition rates from nonCR to CR through ASCT were 15.2%, 21% and 24.1% from 1997 to 2007, 2008 to 2016 and after 2017, respectively. Notably, there was no significant benefit in OS or EFS for tandem transplantation in patients who transitioned from nonCR to CR through the first transplantation (*p* = 0.44 and *p* = 0.1, respectively).

**Fig. 4:**
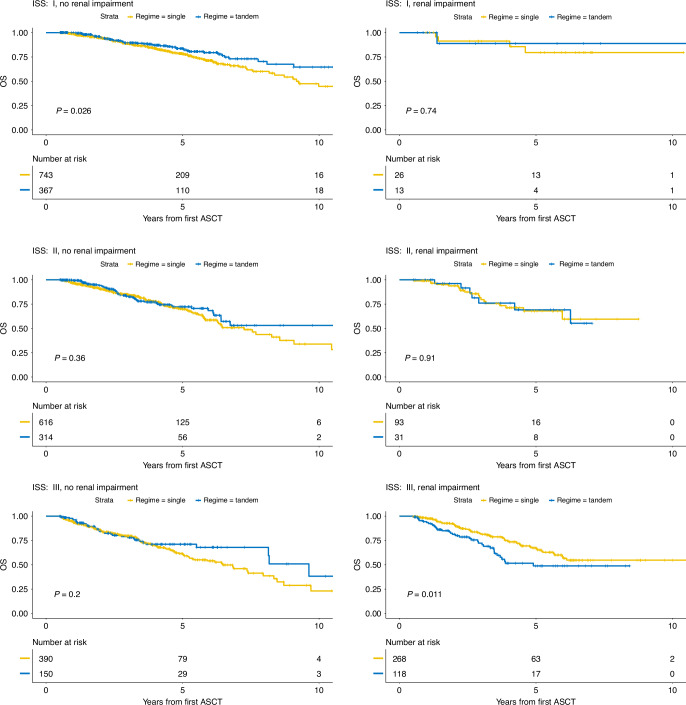
Benefit in OS of tandem ASCT for ISS and renal impairment status based on a 6 month landmark analysis.

Since there is an ongoing discussion about the value of tandem ASCT in certain high-risk populations, we investigated its impact based on ISS and renal impairment. In total, 33.1% of patients with ISS I underwent tandem ASCT, 32.7% with ISS II and 28.9% with ISS III. As for renal impairment, 35.9% and 30.9% received tandem ASCT with Salmon Durie stage A and B, respectively. ISS stage and renal impairment were strongly associated to each other (*p* < 0.001) with only 3.3% of patients with ISS stage I compared to 42.1% of those with ISS stage III having a renal impairment. When comparing the benefits of tandem ASCT on OS, Fig. 4 shows a significant benefit of tandem transplantation for those patients with ISS 1 and no renal impairment (*p* = 0.026). In contrast, results show significantly better OS outcomes for patients with ISS III and renal impairment who underwent single ASCT (*p* = 0.011).

Dose adaption are recommended in patients with renal impairment. Subsequently we analyzed if there was a difference in OS for the conditioning doses based on renal status and found that only those without renal impairment had significantly improved OS receiving a non-reduced dose of 200 mg/m^2^ (*p* < 0.001), while there was no significant improvement in those with renal status B (Supplementary Fig. 2). Additionally, Supplementary Fig. 3 shows that patients with a remission status of PR or worse after induction therapy had a significantly reduced risk of death if conditioned with a melphalan dose of 200 mg/m^2^ compared to 100 mg/m^2^ (HR: 0.62, 0.44–0.86 95% CI, *p* = 0.004).

It has been shown previously that patients with high-risk cytogenetic profiles may benefit from a tandem transplantation regimen [17]. We therefore compared the benefit of different transplantation regimens in overall survival (OS) and event-free survival (EFS) in patients harboring cytogenetic aberrations. However, we found no significant benefit for a tandem regimen in either OS (*p* = 0.99, *p* = 0.58, *p* = 0.97, and *p* = 0.99 for t(4;14) for t(14;16), del17p, and ampl(1q), respectively) or EFS (*p* = 0.17, *p* = 0.77, *p* = 0.93, and *p* = 0.95 for t(4;14), t(14;16), del17p, and ampl(1q), respectively). Similarly, there was no significant difference when combining the cytogenetic profile as “at least one high-risk aberration present” vs. “no aberration present” (*p* = 0.97 for OS and *p* = 0.31 for EFS). The respective survival plots are shown in Supplementary Fig. 4.

In the multivariable analysis, it was shown that younger age was associated in improved OS and EFS outcomes in both single and tandem ASCT regimes. To analyze if tandem ASCT should be performed in elderly patients, we compared regimes in patients aged <=65 years and >65 years. While there was a significant benefit in patients <=65 years of age at first ASCT (*p* = 0.00016 and *p* < 0.0001 for OS and EFS, respectively), no difference was found for elderly patients >65 (*p* = 0.92 and *p* = 0.45 for OS and EFS, respectively). These results are shown in Supplementary Fig. 5.

## Discussion

ASCT achieves high response rates and remains the standard of care for eligible NDMM patients [18]. Across all age groups, ASCT is a viable treatment option, with notable improvements in survival rates, particularly among older patients [11, 19]. Consistent with this trend, our data shows a significant narrowing of survival rate gaps between age groups. Whilst the 5-year survival also significantly increased for elderly patients, the difference across age groups remains substantial at 21%. Our analysis reveals a significant improvement in outcomes for elderly patients, with 5-year OS rising from 39 to 66% in recent years. Despite the absence of anti-CD38-based induction therapies prior to ASCT, these results are comparable to the projected 67% 5-year OS observed in elderly patients receiving daratumumab/lenalidomide/dexamethasone in the MAIA study [20].

Comparing patient cohorts, competing risk introduced through elderly patients dying of non-myeloma related causes must be taken into consideration. With the transplant-eligibility still defining first-line therapy in the era of novel agents, the trends as observed in the presented data continues in a direction that a subset of patients achieve functional cure by having a comparable life expectancy on par with the general population [21]. Our data indicates that very young myeloma patients were not able to equally benefit from the therapeutic breakthroughs of the last decades compared to elderly individuals, even though it shows high survival rates in younger patients that have been reported in previous studies [22–26]. Younger age is associated with macrofocal MM, an uncharacteristic MM presentation, characterized by few bone marrow plasma cells, lytic bone lesions and presence of plasmacytomas and reported to have favorable prognostic features and achieve prolonged survival. The better outcomes in OS were reported in the era of novel agents, as well as in suboptimal regimes, which is in line with the high, but not significantly improving survival times we reported.

Several analyses have been performed in order to identify the optimal candidates for ASCT and especially tandem ASCT. To date, it still remains debatable if patients benefit from a second upfront transplantation [27]. At present, the NCCN panel recommends collecting stem cells for two transplantations in all eligible patients, and considering tandem regimes for patients with high-risk features or those who do not achieve at least VGPR after the first ASCT [18]. The ESMO guideline recommends tandem ASCT for those patients with high risk cytogenetics [28]. While some studies found a benefit of tandem transplantation for the overall cohort, others identified the benefit only for a specific subgroup, and some did not report a difference in treatment regimen [7–9, 29–36]. The phase III GMMG-HD2 trial did not find prolonged EFS or OS in patients treated with tandem SCT. Even though a second transplantation increased the number of responses in VGPR or better, this did not translate to prolonged EFS times in this trial [9, 36]. Achieving a favorable response after ASCT was found to be associated with improved EFS and OS, with several studies reporting that remission status after initial transplantation is the primary clinical discriminator for predicting the benefit of tandem transplantation [8, 31]. Attal et al. [8] found that the effect of single or tandem transplantation on survival differed according to the response achieved after the initial ASCT, with patients not achieving at least VGPR significantly benefitting from a second transplantation. Similarly, the Bologna 96 trial reported no difference in EFS and OS between transplantation regimes for patients with nCR or CR remission status after initial ASCT [31]. Our data confirmed these findings, as our retrospective analyses showed the impact of remission state on the benefit of tandem transplantation. We found that there was no benefit of a tandem ASCT for those patients who achieved CR either before or after initial ASCT. Our data further suggested that patients in VGPR after induction therapy still benefitted from a tandem transplantation, but due to data availability we can only distinguish between CR and nonCR in the treatment stage after first ASCT. The data showed significant transition rates from nonCR after induction therapy to CR after first ASCT, while those patients already in CR after induction therapy kept this status at high rates. Because revised remission criteria were only introduced in 2006 [15], very few VGPR cases are documented in the 1998–2007 time period. It can be hypothesized that the improved transition rates can be attributed to improved VGPR rates following newer induction therapies, which increased from 28.6% in 2008–2016 to 40.5% after 2016. In addition, Lenalidomide maintenance therapy, which was approved in 2017 in Germany, was shown to significantly improve CR and VGPR rates [33]. In this study, we did not have granular information on induction and maintenance regimen surrounding ASCT. However, we tried to address this by including time periods in our analysis to cover central shifts in treatment paradigms in Germany.

In addition to response after initial ASCT, cytogenetic high-risk status has been identified as a factor that might influence the benefit of a tandem transplantation [7, 32–35]. The prospective phase III BMT CTN 0702 StaMINA trial compared three different treatment options following a first ASCT for NDMM: tandem ASCT or Bortezomib, Lenalidomide, Dexamethasone consolidation, both followed by Lenalidomide maintenance or direct Lenalidomide maintenance therapy without consolidation or tandem ASCT. While 6-year EFS was significantly improved in high-risk cytogenetics patients receiving tandem transplantation, there was no difference in 6-year OS and EFS in the overall population [33–35]. The EMN02/HO95 study also compared transplantation regimes and found significantly improved OS and EFS outcomes for patients who received tandem ASCT in the overall cohort and the HR for disease progression or death favouring tandem over single ASCT was not as high in the subgroup of patients with standard-risk cytogenetics as it was in high-risk patients [32]. Gagelmann et al. [7] showed that tandem transplantation in t(4;14) patients was associated with improved EFS. There was no difference in OS for t(4;14) and no difference in EFS or OS for del(17p). In the presented study we did not find an advantage of a tandem regime for patients with a high-risk cytogenetic profile; however, cytogenetic data was documented for only a small portion of our dataset.

Notably, we found that patients with ISS III risk status and renal impairment have significantly lower survival rates in the cohort receiving tandem transplantation. Because Melphalan is partially renally cleared, patients with renal impairment were shown to experience more toxicity [37] and it was described before that in renal failure patients, tandem SCT did not improve OS or EFS [38]. Badros et al. [38] further reported a treatment-related mortality of 6% after the first compared to 13% after the second ASCT. Patients with renal failure experienced high rates of severe toxicities, including infections, gastrointestinal complications, mucositis, as well as pulmonary and neurological complications. Among these, mucositis and pulmonary events were significantly more frequent in patients receiving 200 mg/m^2^ of melphalan conditioning compared to 140 mg/m^2^ preceding the initial ASCT. Additionally, dialysis-dependent patients exhibited a significantly higher incidence of pulmonary, neurological, and skin-related complications. Our study is limited in the aspect that we are not able to distinguish between mild, moderate, severe, and dialysis-dependant renal impairment, as well the incomplete documentation of conditioning doses for second ASCTs. However, ISS III is associated with increased severity in renal impairment [39], which could indicate that the added toxicity through a tandem regime is especially burdensome in those patients with severe renal impairment.

In summary, outcomes for transplanted patients across all age groups have improved significantly over the last decades, especially in older patients. In our retrospective analysis, we showed that while most patients did benefit from tandem transplantation, those at older age or who achieved CR after initial ASCT did not have an advantage from a second ASCT, and those with ISS III and renal impairment even have significantly decreased survival rates and should not be considered for tandem ASCT.

## Supplementary information

Supplement

## Data Availability

Due to the scope of patient agreements, data cannot be shared to third parties by the authors. The data is accessible by application to the DRST.
