# Supplementary material for: Single versus tandem autologous stem cell transplantation in newly diagnosed multiple myeloma
Source: Bone Marrow Transplant. 2024 Dec 5;60(3):335–45. doi: 10.1038/s41409-024-02490-1 (PMC11893441; doi:10.1038/s41409-024-02490-1)
Supplement: Supplementary file 1 — Supplement [file 41409_2024_2490_MOESM1_ESM.pdf]

Supplementary Table 1: Included study patients

DRST Principal investigator	Hospital	Patients
Professor Peter Dreger	Universitätsklinikum Heidelberg	1383 patients
Dr. Hans-Jürgen Salwender	Aslepios Klinik Altona	491 patients
Professor Guido Kobbe	Universitätsklinikum Düsseldorf	466 patients
Professor Robert Zeiser	Universitätsklinikum Freiburg	437 patients
Professor Uwe Platzbecker	Universitätsklinikum Leipzig	410 patients
Professor Igor Wolfgang Blau	Charité - Universitätsmedizin Berlin	394 patients
Dr. Daniel Teschner	Universitätsklinikum Würzburg	373 patients
Dr. Christoph Kimmich	Klinikum Oldenburg AöR	373 patients
Dr. Elisa Sala	Universitätsklinikum Ulm	367 patients
Professor Wolfgang Bethge	Universitätsklinikum Tübingen	294 patients
Dr. Anke Morgner	Klinikum Chemnitz	290 patients
Professor Matthias Stelljes	Universitätsklinikum Münster	284 patients
Professor Matthias Edinger	Universitätsklinikum Regensburg	248 patients
Professor Inken Hilgendorf	Universitätsklinikum Jena	244 patients
Dr. Richard Ratei	Helios Klinikum Bad Saarow	235 patients
Professor Roland Schroers	Ruhr Universität Bochum	228 patients
Professor Friedrich Stölzel	Universitätsklinikum Kiel	216 patients
Dr. Judith Niederland	Helios Klinikum Berlin-Buch	212 patients
Dr. Kerstin Schäfer-Eckart	Klinikum Nürnberg Nord	204 patients
Professor Andreas Burchert	Universitätsklinik Marburg	204 patients
Dr. Ute Wieschermann	HELIOS Klinikum Duisburg GmbH	193 patients
Dr. Martin Kaufmann	Robert-Bosch-Krankenhaus Stuttgart	190 patients
Professor Johannes Schetelig	Universitätsklinikum Carl Gustav Carus a. d. TU Dresden	190 patients
Dr. Gesine Bug	Universitätsklinikum Frankfurt (Main)	189 patients
Dr. Miriam Ahlborn	Städt. Klinikum Braunschweig	184 patients
Professor Christof Scheid	Universitätsklinikum Köln	180 patients
Dr. Eva Wagner-Drouet	Universitätsmedizin Mainz	175 patients
Dr. Denise Wolleschk	Universitätsklinikum Magdeburg	172 patients
Dr. Mareike Verbeek	Klinikum rechts der Isar der TU München	165 patients
Dr. Stefan Kaun	Klinikum Bremen-Mitte	161 patients
Dr. Su Hyeon Kim	Klinikum Ernst-von-Bergmann Potsdam	159 patients
Professor Matthias Eder	Med. Hochschule Hannover	143 patients
Dr. Tobias Holderried	Universitätsklinikum Bonn	140 patients
Professor Ahmet Elmaagacli	Asklepios Klinik St. Georg Hamburg	138 patients
Professor Christoph Schmid	Universitätsklinikum Augsburg	118 patients
Islam Hussein Mohamed	Klinikum Osnabrück	115 patients
Professor Mark Ringhoffer	Städt. Klinikum Karlsruhe gGmbH	115 patients
Dr. Julia Winkler	Universitätsklinikum Erlangen	113 patients
Dr. Thomas Schroeder	Universitätsklinikum Essen	111 patients
Professor Edgar Jost	Universitätsklinik RWTH Aachen	109 patients
Dr. Mareike Dürholt	KEM - Kliniken Essen-Mitte Evang. Krankenhaus Essen-Werden	102 patients
Professor Gerlald Wulf	Universitätsklinik Göttingen	101 patients
Professor Lutz P. Müller	Universitätsklinikum Halle (Saale)	100 patients
Professor Ulrich Graeven	Kliniken Maria Hilf Mönchengladbach	97 patients
Dr. Stefan Klein	Universitätsmedizin Mannheim	97 patients
Professor Gerald Illerhaus	Katharinenhospital Stuttgart	95 patients
Professor William Krüger	Universitätsklinikum Greifswald	94 patients
Andrea Stoltefuß	Ev. Krankenhaus Hamm	86 patients
Professor Carsten Bokemeyer	UKE-II Med. Klinik Hamburg	84 patients
Professor Nicolaus Kröger	Universitätsklinikum Hamburg-Eppendorf	80 patients
Dr. Karin Schmitz	Ev. Diakonie-Krankenhaus	78 patients
Professor Angela Krackhardt	Malteser Krankenhaus Flensburg	77 patients
Professor Herbert Gottfried Sayer	Helios Klinikum Erfurt	76 patients
Dr. Frederike Wortmann	Universitätsklinikum Schleswig-Holstein / Campus Lübeck	76 patients
Dr. Susanne Hain	St. Marien-Krankenhaus Siegen	75 patients
Barbara Ritter	Klinikum Kassel	73 patients
Dr. Xaver Schiel	Klinikum Harlaching München	73 patients

Dr. Jakob Maucher	Diakonie-Klinikum Stuttgart	59 patients
Professor Doris Kraemer	Kath. Krankenhaus Hagen	57 patients
Dr. Johannes Lakner	Universitätsmedizin Rostock	54 patients
Dr. Arne Brecht	DKD HELIOS Klinik Wiesbaden	51 patients
Dr. Johanna Tischer	Klinikum der Universität München -Großhadern	47 patients
Dr. Dirk Niemann	Gemeinschaftsklinikum Mittelrhein Koblenz	47 patients
Professor Michael Kiehl	Klinikum Frankfurt (Oder)	44 patients
Dr. Ralf Georg Meyer	Gem. Transpl. Dortmund-Mitte	44 patients
Dr. Sarunas Bagdonas	Klinikum Lippe Lemgo	42 patients
Professor Wolfgang Knauf	Agaplesion Bethanien Krankenhaus Frankfurt (Main)	41 patients
Professor Marcus Hentrich	Rotkreuzklinikum München	41 patients
Professor Gerhard Held	Westpfalz-Klinikum Kaiserslauter	38 patients
Dr. Tobias Bartscht	Helios Klinikum Schwerin	37 patients
Dr. Friedhelm Krebbel	Sana Klinikum Hameln	37 patients
Dr. Moritz Lesse	Dr. Horst-Schmidt-Kliniken Wiesbaden	37 patients
Dr. Martin Hoffmann	Klinikum Ludwigshafen	27 patients
Professor Roland Repp	Städtisches Krankenhaus Kiel	21 patients
Dr. Detlev Kohl	Ammerland-Klinik Westerstede	16 patients
Professor Markus Schaich	Rems-Murr-Kliniken Winnenden	15 patients
Dr. Eva-Bettina Zinngrebe	Ev. Klinikum Bethel Bielefeld	14 patients
Dr. Achim Meinhardt	Diakoniekrankenhaus Rotenburg (Wümme)	14 patients
Professor Axel Fauser	Klinikum für KMT Idar-Oberstein	14 patients
Dr. Wolfgang Schmidt	Städt. Klinikum Frankfurt-Höchst Frankfurt (Main)	13 patients
Professor Sebastian Bauer	Uni - Klinik f. Innere Med (Tumorf.) Essen	13 patients
Dr. Tobias Gaska	Brüderkrankenhaus St. Josef Paderborn	12 patients
Dr. Martin Schmidt-Hieber	Carl-Thiem-Klinikum Cottbus	9 patients
Dr. Axel Florschütz	Städt. Klinikum Dessau	8 patients
Professor Fuat Oduncu	Klinikum Innenstadt München	7 patients
Dr. Manfred Planker	Klinikum Krefeld	7 patients
Professor Paul La Roseée	Schwarzwald-Baar Klinikum Villingen-Schwenningen	5 patients
Professor Hans Josef Weh	Franziskus Hospital Bielefeld	4 patients
Professor Dirk Strumberg	Marienhospital Herne	3 patients
Professor Alexander Kiani	Klinikum Bayreuth	3 patients
Professor Hans-Jürgen Mergenthaler	Bürgerhospital Stuttgart	2 patients
Dr. Mohammad Wattad	Klinikum Hochsauerland Meschede	1 patient
Dr. Gero Massenkeil	Städt. Krankenhaus Gütersloh	1 patient
Dr. Daniela Dörfel	Klinikum Hannover	1 patient

Supplementary Table 2: Packages and versions used in the analysis

Environment	Package	Version
R	dplyr	1.1.3
R	ggplot2	3.4.4
R	ggpubr	0.6.0
R	ggsci	3.0.0
R	scales	1.2.1
R	purrr	1.0.2
R	tidyr	1.3.0
R	stringr	1.5.1
R	survival	3.5.7
R	survminer	0.4.9

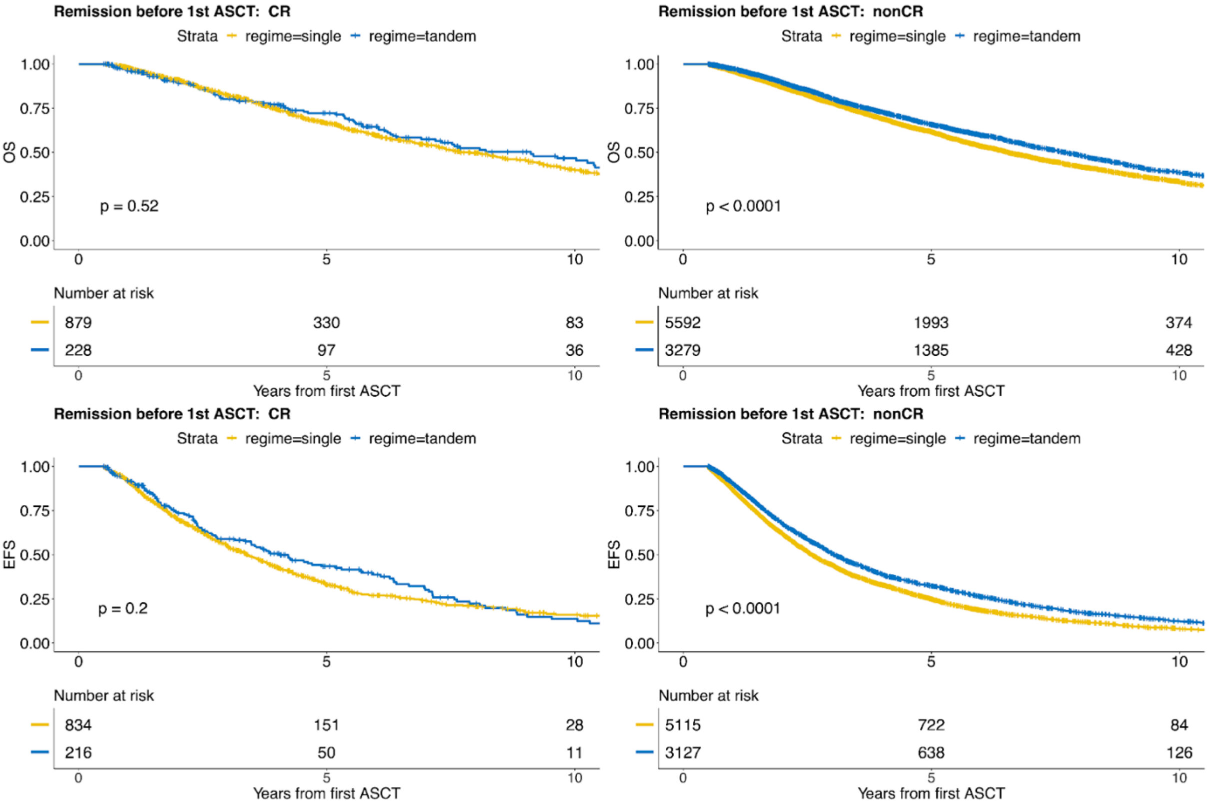

Supplementary Figure 1: Benefit of tandem transplantation for remission after induction therapy based on a 6 month landmark analysis on OS and EFS

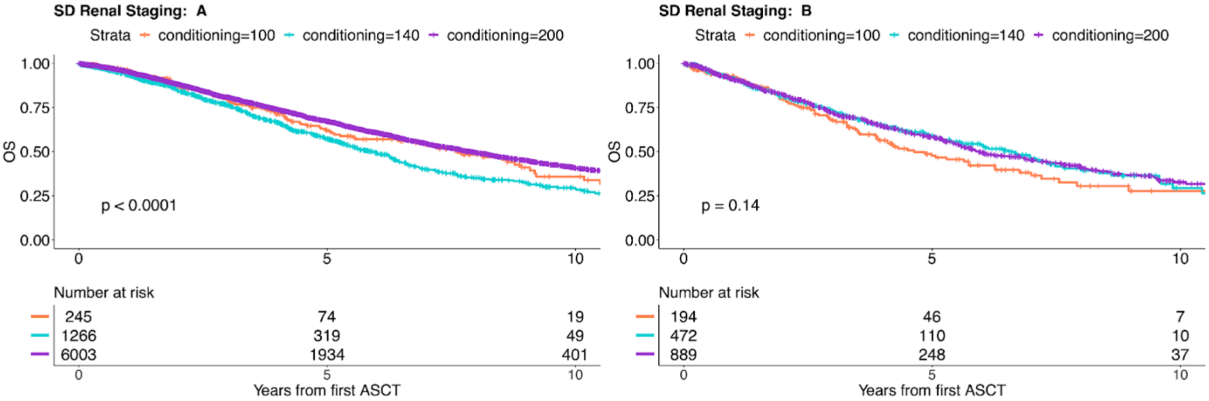

Supplementary Figure 2: Benefit of conditioning doses for renal staging on OS

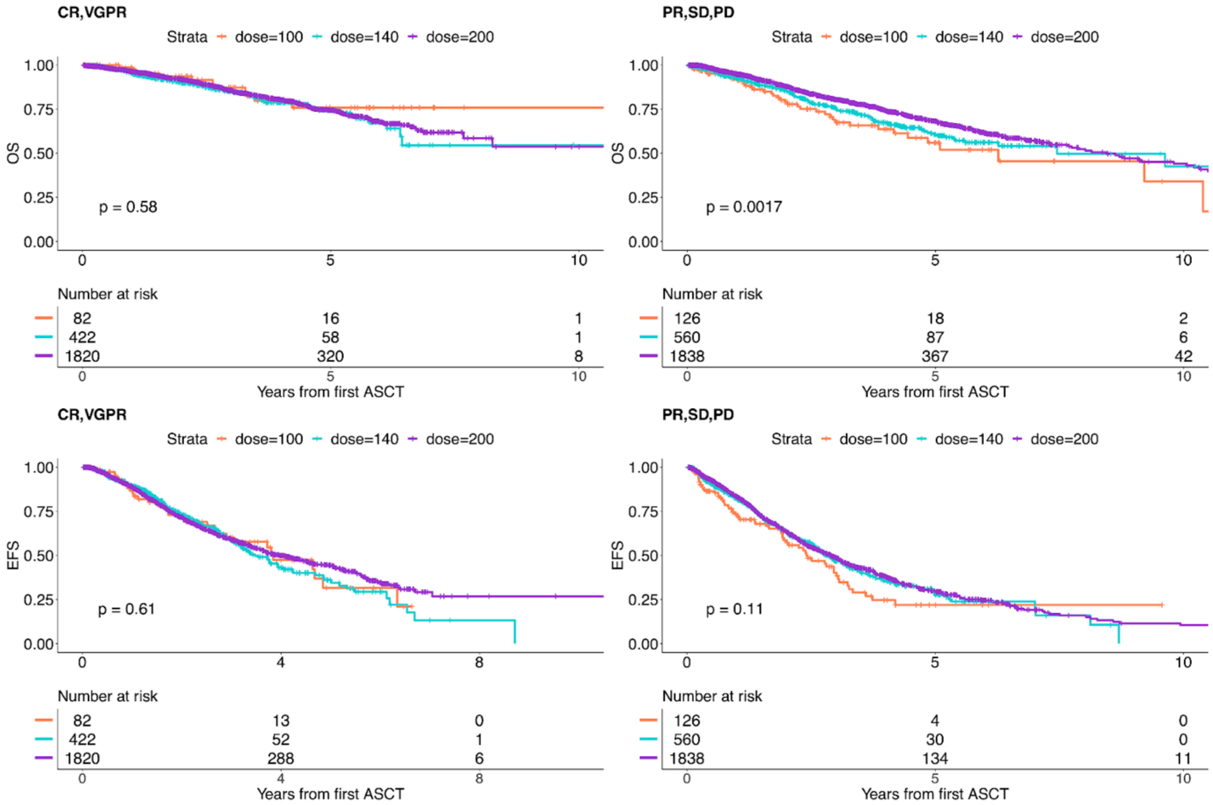

Supplementary Figure 3: Benefit in OS and EFS of upfront conditioning doses for remission status after induction therapy of CR/VGPR vs. PR/SD/PD

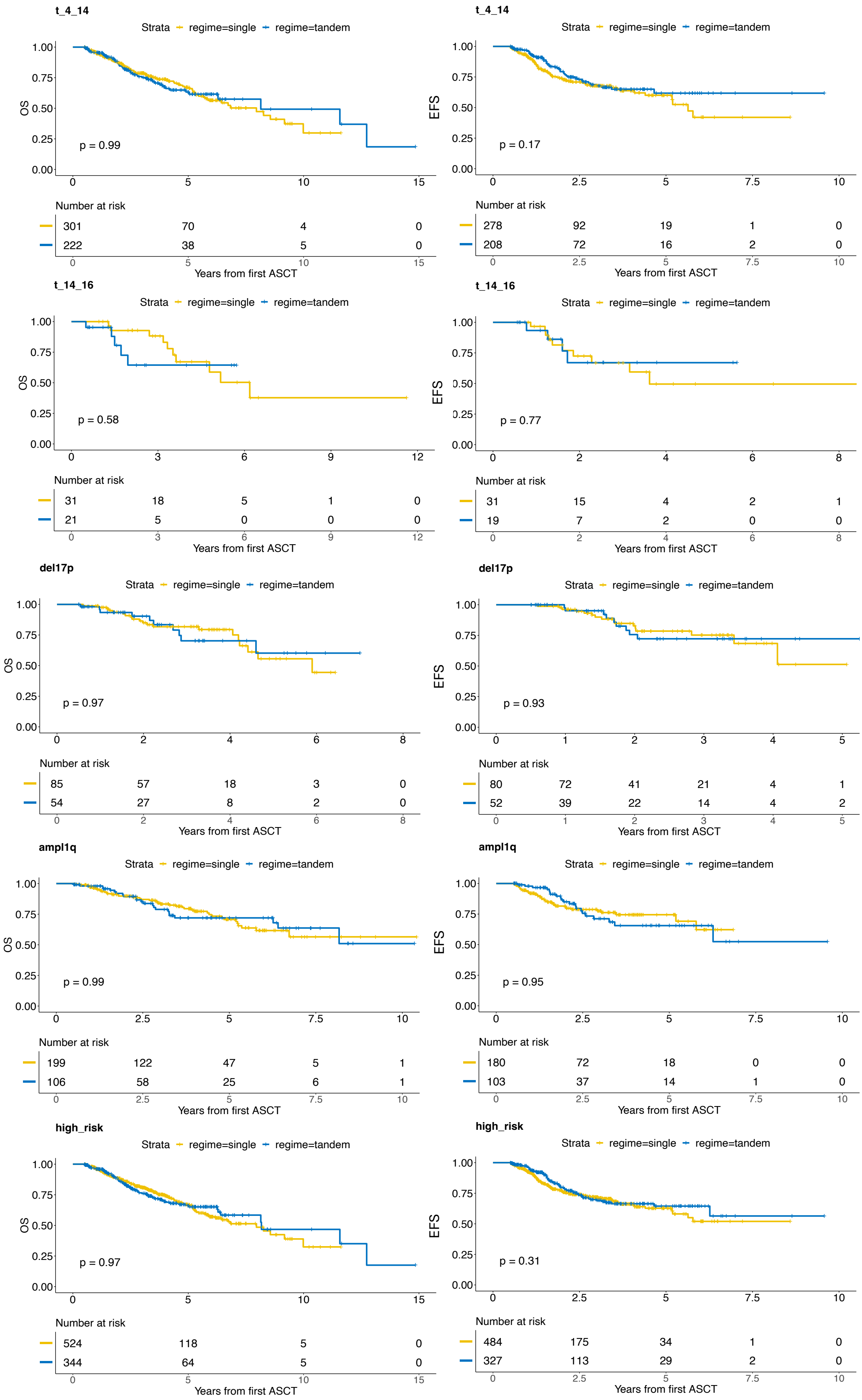

Supplementary Figure 4: Benefit of tandem transplantation for high-risk cytogenetics on OS and EFS

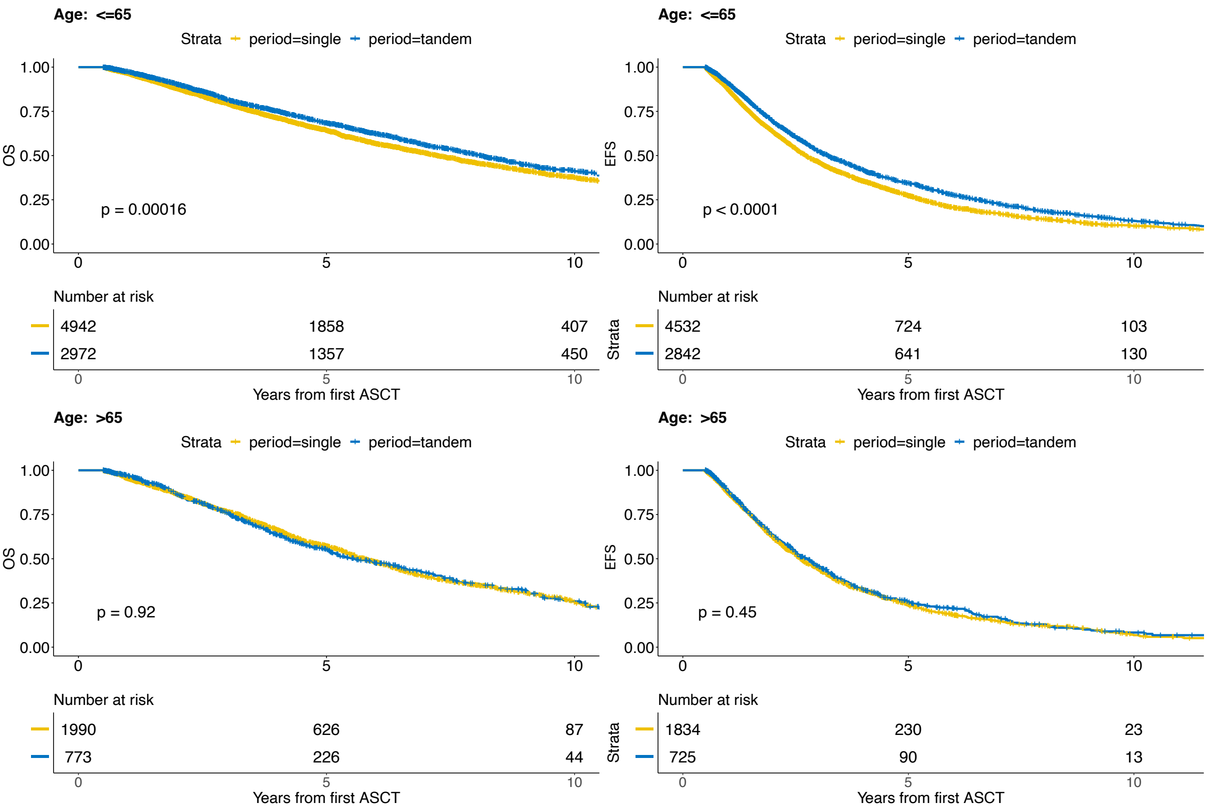

Supplementary Figure 5: Benefit of tandem transplantation for patients according to patients aged <=65 vs. >65 on OS and EFS
